# Numerical and experimental analyses for the improvement of surface instant decontamination technology through biocidal agent dispersion: Potential of application during pandemic

**DOI:** 10.1371/journal.pone.0251817

**Published:** 2021-05-19

**Authors:** Paulo Roberto Freitas Neves, Turan Dias Oliveira, Tarcísio Faustino Magalhães, Paulo Roberto Santana dos Reis, Luzia Aparecida Tofaneli, Alex Álisson Bandeira Santos, Bruna Aparecida Souza Machado, Fabricia Oliveira Oliveira, Leone Peter Correia da Silva Andrade, Roberto Badaró, Luis Alberto Brêda Mascarenhas

**Affiliations:** 1 SENAI CIMATEC, National Service of Industrial Learning–SENAI, Computational Modeling and Industrial Technology, University Center SENAI/CIMATEC, Salvador, Bahia, Brazil; 2 SENAI CIMATEC, National Service of Industrial Learning–SENAI, SENAI Institute of Innovation (ISI) in Health Advanced Systems (CIMATEC ISI SAS), University Center SENAI/CIMATEC, Salvador, Bahia, Brazil; VIT University, INDIA

## Abstract

The transmission of SARS-CoV-2 through contact with contaminated surfaces or objects is an important form of transmissibility. Thus, in this study, we evaluated the performance of a disinfection chamber designed for instantaneous dispersion of the biocidal agent solution, in order to characterize a new device that can be used to protect individuals by reducing the transmissibility of the disease through contaminated surfaces. We proposed the necessary adjustments in the configuration to improve the dispersion on surfaces and the effectiveness of the developed equipment. Computational Fluid Dynamics (CFD) simulations of the present technology with a chamber having six nebulizer nozzles were performed and validated through qualitative and quantitative comparisons, and experimental tests were conducted using the method Water-Sensitive Paper (WSP), with an exposure to the biocidal agent for 10 and 30 s. After evaluation, a new passage procedure for the chamber with six nozzles and a new configuration of the disinfection chamber were proposed. In the chamber with six nozzles, a deficiency was identified in its central region, where the suspended droplet concentration was close to zero. However, with the new passage procedure, there was a significant increase in wettability of the surface. With the proposition of the chamber with 12 nozzles, the suspended droplet concentration in different regions increased, with an average increase of 266%. The experimental results of the new configuration proved that there was an increase in wettability at all times of exposure, and it was more significant for an exposure of 30 s. Additionally, even in different passage procedures, there were no significant differences in the results for an exposure of 10 s, thereby showing the effectiveness of the new configuration or improved spraying and wettability by the biocidal agent, as well as in minimizing the impact caused by human factor in the performance of the disinfection technology.

## Introduction

COVID-19, a disease characterized by a severe acute respiratory syndrome and caused by coronavirus-2 (SARS-CoV-2), first occurred in Wuhan, China and spread worldwide in a few weeks [1–4]. On March 11, 2020, the World Health Organization (WHO) declared COVID-19 to be a pandemic [5–7]. The United States, India, and Brazil are the three countries with the highest number of reported cases, representing 51% of the total SARS-CoV-2 infections worldwide [8].

Although there have been warnings about the threat of viruses that cause respiratory diseases [9], the SARS-CoV-2 virus has spread at an unprecedented rate, and there is an urgent need to formulate various approaches to face this pandemic [10–13]. The transmission of SARS-CoV-2 can occur through direct contact with contaminated surfaces or by air, mainly through direct contact with contaminated people and biological secretions [14–20]. Since the beginning of the pandemic, different countries adopted different challenging measures to reduce infection rates and avoid the collapse of health systems [21, 22]. A variety of non-pharmaceutical interventions were adopted, such as complete regional blockages, closing of non-essential activities/commerce, mass testing of the population, quarantine measures, tracking the infected, construction of hospitals for the treatment of COVID-19, and development of new disinfection technologies [23, 24]. Transmission through contact with contaminated surfaces or objects has been described as an important form of transmissibility [25, 26], which has even been demonstrated for SARS-CoV-2 [27, 28]. The search and evaluation of the efficacy of some biocidal agents and technologies that disinfect contaminated environments and surfaces is based on previous studies, mainly with related viruses, such as SARS-CoV (Severe Acute Respiratory Syndrome Coronavirus) and MERS (Middle Eastern Respiratory Syndrome Coronavirus) [29–31]. To date, there is no specific treatment for SARS-CoV-2, however, since the beginning of the pandemic, a race against time has begun to make a vaccine for disease prevention available quickly. Currently, the vaccination against COVID-19 could be achieved mainly through emergency approvals for use by the regulatory agencies in each country. These approvals are mechanisms to facilitate the use of medicines in emergency situations, that may be used (even if not officially approved), but if certain statutory criteria have been met, including for example, that there are no other available alternatives [32]. The disinfection measures are also part of this race for preventive methodologies, especially at the time when vaccines were not yet available and also during the vaccination process, since this process has not yet been able to cover the entire population.

Disinfection measures can help inhibit the transmissibility of the virus, provided that they are safe for use. When it comes to transmissibility, it is important to emphasize that several studies have been bringing more and more data about the importance of disinfection measures, given the virus’ ability to stay alive in the environment for considerable periods of time. The environmental risks associated with COVID-19, such as the waste generated and contaminated by the virus, have been receiving great attention from researchers [33]. Hospitals, home care and quarantine facilities, for example, are generating a large amount of waste. Face masks, personal protective equipment (PPE), nitrile gloves, disposable caps, among others, despite the importance of their use for individual protection, are considerable sources of contamination. And for this reason, the concern with the disinfection measures applied to these residues is also important in the context of the pandemic [34, 35]. Still, it is worth to note that the search for new disinfection measures can not only be applied to the current pandemic of COVID-19, but also to other situations that may occur in the future, since the growth in the number of infections associated with new diseases may become an increasingly frequent factor in the future.

Since the beginning of the pandemic, new technologies and protocols have been developed with the purpose of exercising microbial control efficiently, thereby targeting a reduction in the infection rate [36, 37]. Here, we discuss technologies that have been adopted to help combat contamination by SARS-CoV-2. For example, previous studies have demonstrated the benefits of using ultraviolet light devices for disinfecting hospital environments [38], portable devices with spray systems for surface decontamination [39], and disinfection chambers with different biocidal agents [40]. These new developments have been shown to assist in the control of microbial load based on evidence from tests on different microorganisms and have mainly been applied in nosocomial environments, which are one of the main public health problems worldwide [41, 42] and therefore have considerable potential for application during and after the COVID-19 pandemic.

An equipment was recently developed to disperse a solution containing biocidal materials for the instant disinfection of personal protective equipment (PPE), worn by healthcare workers when leaving hospital areas intended for treatment of patients with SARS-CoV-2 in some of the reference hospitals for the treatment of COVID-19 in Brazil. The evaluation of its effectiveness, safety, and acceptance among professionals was carried out through experimental tests using previously contaminated surfaces and qualitative analysis of the deposition of particles in the study regions [43], as well as by collecting information using structured questionnaires involving more than 400 professionals who have used it. This equipment, called disinfection chamber, was designed as an alternative strategy to the current possibilities of protection against COVID-19, and its objective is to decontaminate potential surfaces, such as PPE, which may contribute to the high transmission of SARS-CoV-2 among health professionals during the process of doffing step. It is worth mentioning that the use of this equipment may also be applied in other situations, not only in nosocomial environments, increasing the scope of protection to individuals.

In addition to the disinfection properties of the solution, the effectiveness of this technology is also dependent on the configuration and form of use of the equipment. The nozzle arrangement and passage procedure can influence the wettability of the surfaces and thus the disinfection performance [44].

Numerical simulations have been shown to be useful for assessing air flow and particulate trajectory, where it is important to understand the physical phenomenon for medical and research purposes [45–48]. Some studies have shown the development of disinfection chambers using CFD and have illustrated validation techniques using such type of simulations [44, 49]. Hence, CFD can be an interesting alternative for evaluating existing technologies or new propositions [49, 50].

Thus, this study evaluated the performance of a disinfection chamber designed for instantaneous dispersion of biocidal agent solution. We proposed the necessary adjustments in the configuration to improve the dispersion on surfaces and the effectiveness of the developed equipment. Therefore, different exposure times, passage procedures inside the chamber, and nebulizer nozzle configurations were evaluated to improve the application of the technology as an additional barrier against contamination by SARS-CoV-2.

## Materials and methods

**Fig 1:**
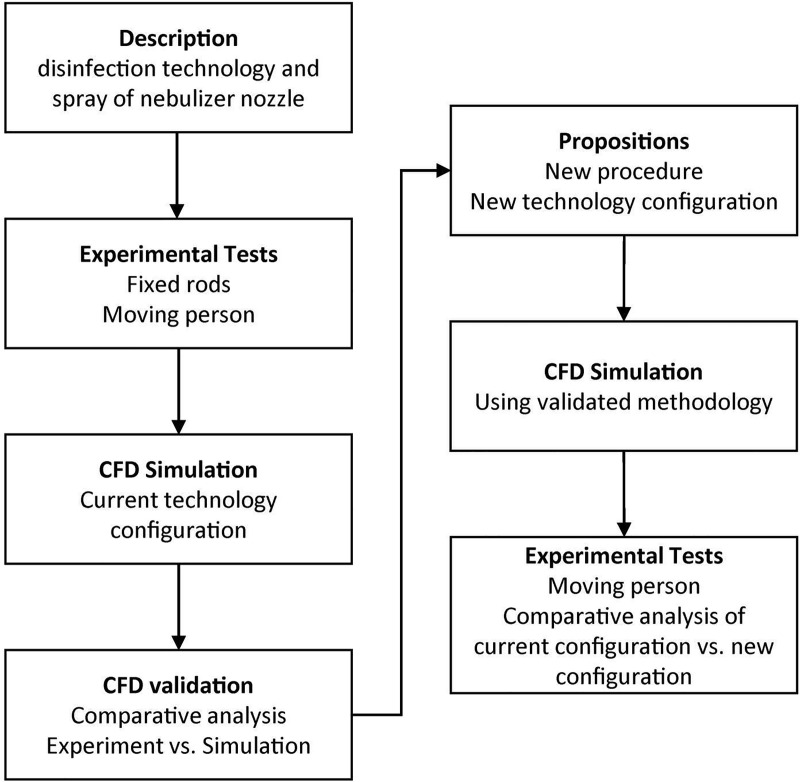
General scheme of the methodology used in this study to evaluate the performance of the disinfection technology (chamber) in relation to the dispersion of the biocidal agent.

Experiments and numerical simulations were used to test a previously developed disinfection chamber [43]. The experimental tests were conducted with the objective of providing input information for the simulations and comparative analyses, applying flow measurement methods, thermography, and water sensitive paper (WSPs) [33, 35, 51–55]. The simulations were carried out in two stages. The first stage involved the nebulizer nozzle to gain a proper understanding of the dispersion behavior in the nozzle, and the second stage was related to the effectiveness of the biocidal agent dispersion in the disinfection system (chamber). Fig 1 presents the scheme of the methodology applied in this study to evaluate the dispersion of the biocidal agent solution (aqueous solution of sodium hypochlorite with a concentration of up to 0.25%).

### Disinfection technology (chamber)

**Fig 2:**
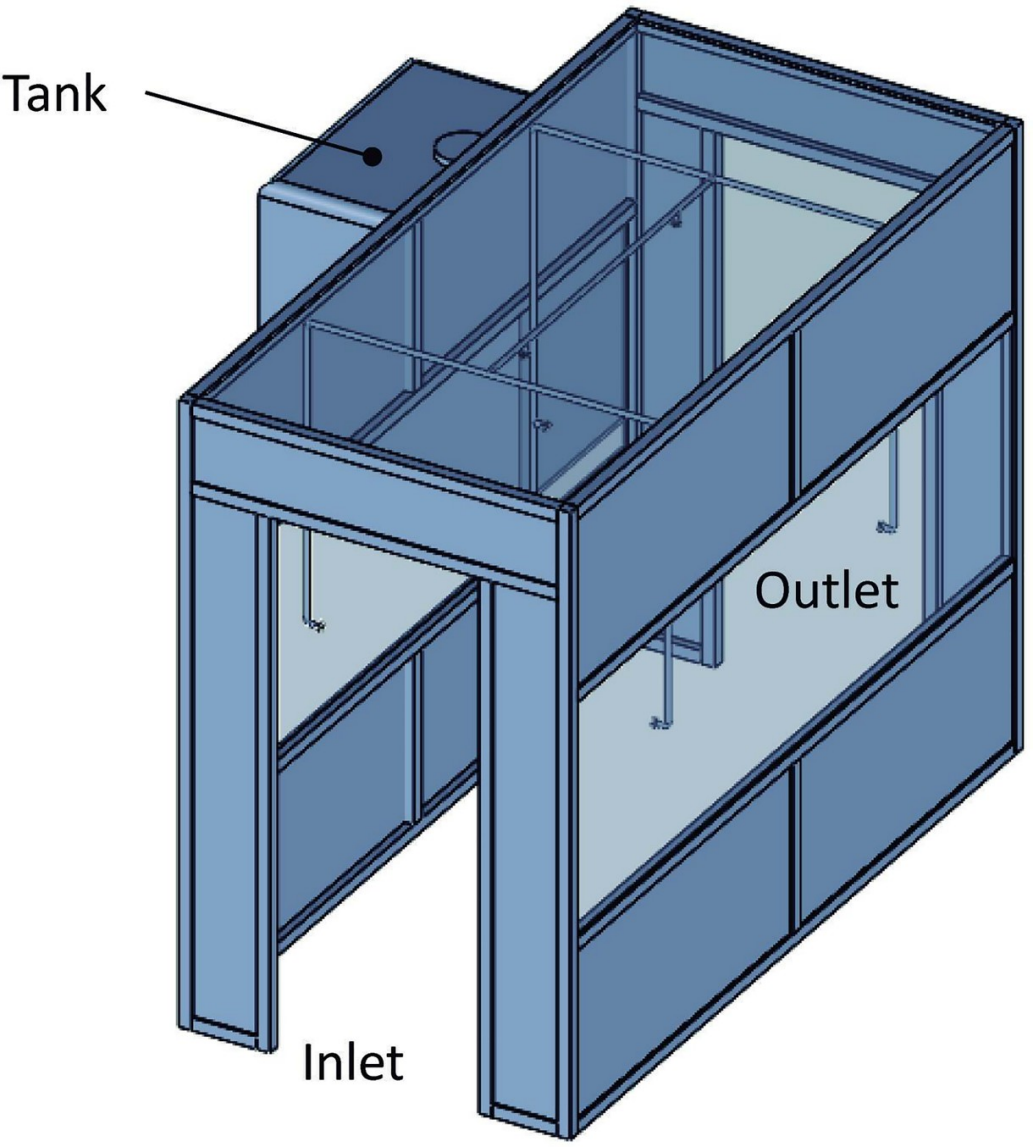
Representation of the disinfection chamber developed for instant decontamination of surfaces using biocidal agents.

The disinfection chamber used in this study was developed as a modular structure, with the dimensions 2,4 x 1,5 x 3,0 m (HxWxL), composed of aluminum profiles and closure in material acrylic and PVC (polyvinyl chloride). Internally, the chamber had a nebulization system composed of six nebulizer nozzles installed on the sides (horizontal position), ceiling (vertical position), a water filter, submerged pump, and storage tank with a capacity of 1000 L (Fig 2). A control unit was responsible for activating the system, with the presence sensor for activation of the nebulizer nozzles if an individual passed through the chamber [56, 57].

### Characterization of the spray formed by the nebulizer nozzle

The spray formed by the nebulizer nozzle had different angles, droplet sizes, and flow rates. These variables were used as input data in the numerical simulations [47, 58–60]. Hence, the nozzle volumetric flow was determined by collecting three samples of solution volumes (one minute per sample) and then calculating the average volumetric flow.

The angle formed by the spray was measured by collecting thermal images of the spray using a thermographic camera, according to the methodology proposed by Jiao et al. [60] obtained images to visualize the spray due to the difference between the temperatures of the fluid and the internal walls of the disinfection chamber. For the measurement of this parameter, six thermal images were captured at vertical and horizontal positions of the nozzle at different instants.

The measurement of the spray particle size was performed thrice, collecting droplets of the spray formed by the nebulizer nozzle using WSPs positioned in front of the nozzle at a distance of 65 mm. After collection, the WSPs were read using WSP reading technology (DropScope, Brazil). Thus, it was possible to detect overlapping droplets [61], with a minimum recorded size of 24.18 μm. The obtained data were compiled and adjusted to a Rosin–Rammler distribution function, suitable for representing the distribution of particles and droplets [62–64]. This distribution is characterized by a scale factor *d*_*e*_ and form factor *γ*.

### Wettability analysis

**Fig 3:**
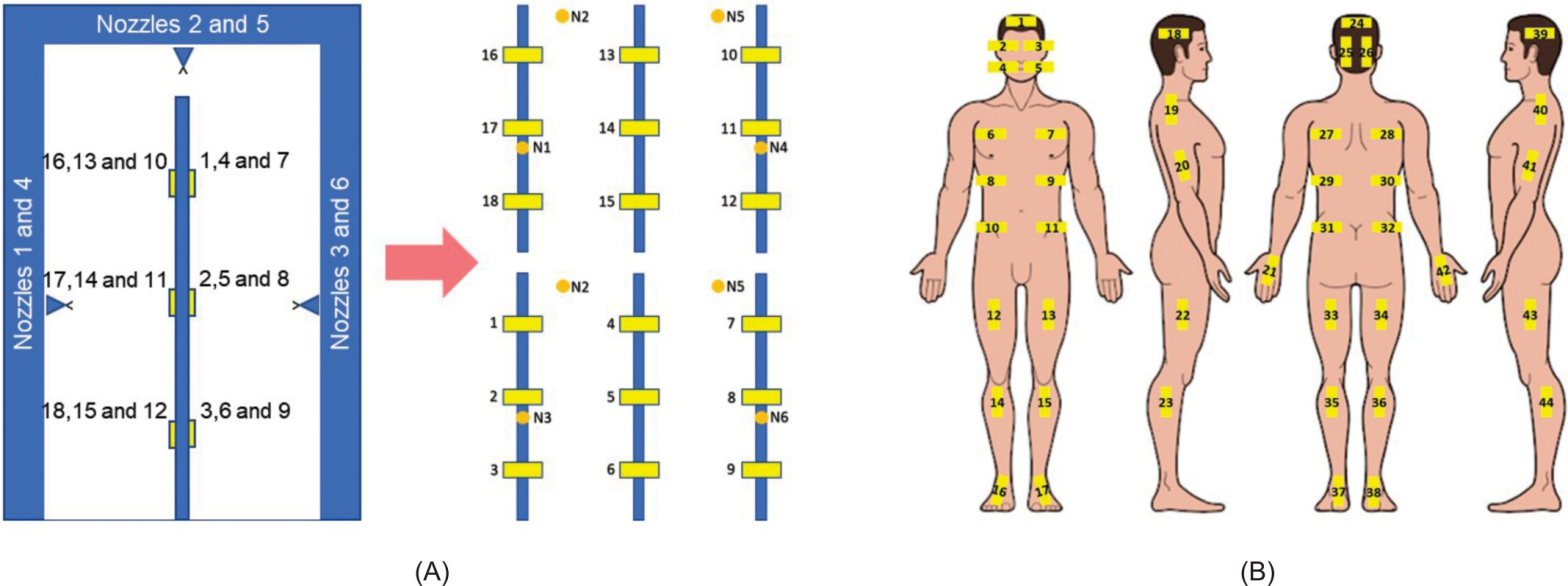
Illustration of the regions where WSPs were applied. (A) Experiment with the rods in the central area of the chamber; (B) experiment with the properly dressed individual in PPE.

Wettability analysis was performed using the WSP method in two different ways. Initially, 18 WSPs were applied to rods fixed in the ranges identified as 1, 2, and 3 arranged in the central area of the disinfection chamber, as shown in Fig 3A. Thus, the WSPs were exposed for a period of 10 s and then analyzed using DropScope technology, and the percentage of wet area of each WSP was calculated. In the second step, 44 WSPs were applied to different regions of the body of a person previously dressed with PPE (cap, glasses, mask, gloves, coat, and shoes) (Fig 3B) in order to qualitatively assess the wettability of the surfaces in different regions of the PPE during exposure in the disinfection chamber. For this analysis, two exposure times to the biocidal agent, 10 and 30 s, were used, where the individual made a 360° rotation during the passage through the chamber. Additionally, for 10 s exposure, the direct passage through the chamber was also evaluated (without turning in the center). All experimental tests for wettability analysis were performed twice.

### Computational fluid dynamics

#### Simulation of nebulizer nozzle

**Fig 4:**
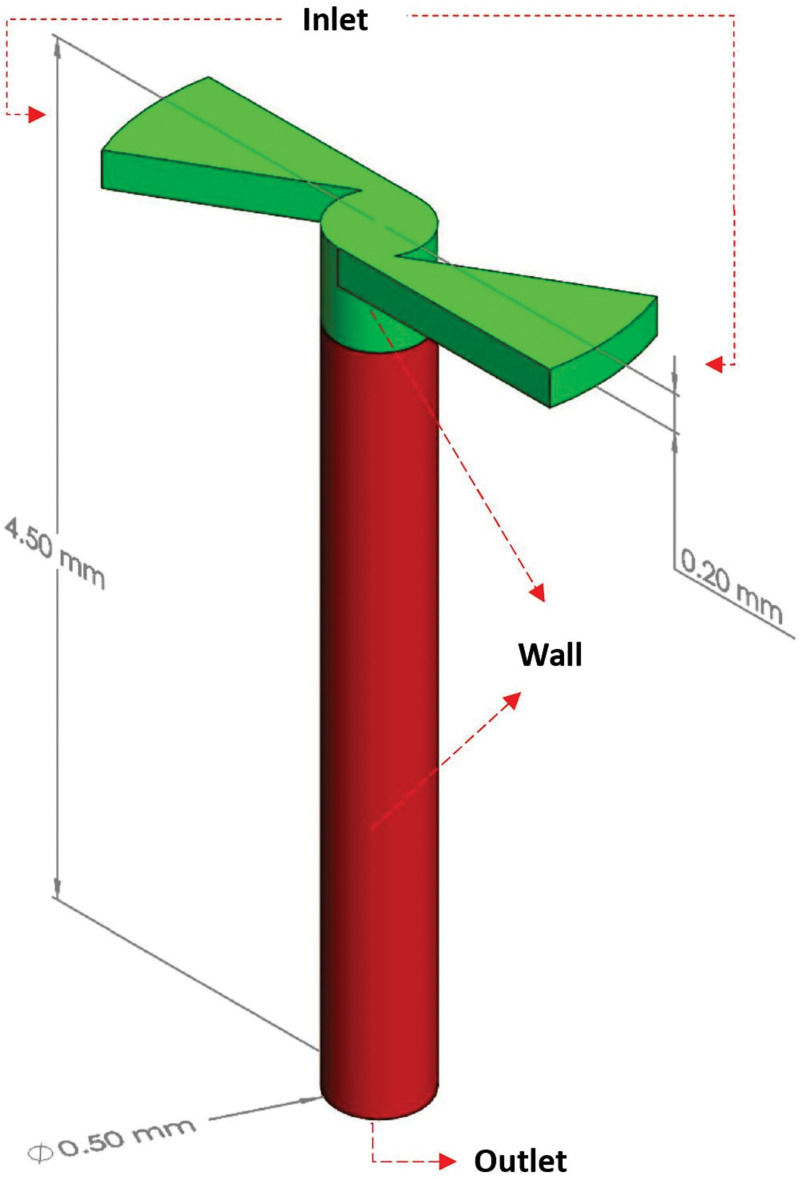
Computational domain illustration of the nebulizer nozzle.

To study the flow in the disinfection chamber, it was necessary to conduct simulations of the nebulizer nozzles to determine the speed of injection of the droplets. Here, ANSYS CFX 17.1 software was used to simulate the flow of nozzles. The modeled computational domain presents the geometric characteristics in which there is continuous flow of the solution before it leaves the nebulizer nozzle, as shown in Fig 4. The green region represents the internal part of the nebulizer nozzle, and the red extension is modeled for numerical stability.

The Eulerian approach through the finite volume method [65] was adopted to obtain the solution of the equations that describe the flow through the continuous medium of the nozzle. The method in question solves the equations of conservation of mass (continuity) and momentum (Navier-Stokes), described by Eqs 1–4:

$$\frac{\partial \rho }{\partial t}+div(\rho \vec{u})=0$$ (1)

$$\frac{\partial (\rho u)}{\partial t}+div(\rho u\vec{u})=-\frac{\partial p}{\partial x}+div(\mu \;grad\;u)+S_{Mx}$$ (2)

$$\frac{\partial (\rho v)}{\partial t}+div(\rho v\vec{u})=-\frac{\partial p}{\partial y}+div(\mu \;grad\;v)+S_{My}$$ (3)

$$\frac{\partial (\rho w)}{\partial t}+div(\rho w\vec{u})=-\frac{\partial p}{\partial z}+div(\mu \;grad\;w)+S_{Mz}$$ (4)

where p is the pressure; t is time; ρ is density; x, y, and z are the three Cartesian directions; u, v, and w are the speeds in the x, y, and z directions, respectively; $\vec{u}$ is the three-dimensional velocity vector; μ is the viscosity of the fluid; S_Mx_, S_My_, and S_Mz_ are the source terms of momentum in the directions x, y, and z, respectively. n the present simulation, the Shear Stress Transport (SST) k–ω turbulence model was used. The application of this model requires the solution of two more transport equations (one for turbulent kinetic energy, k, and another for turbulent frequency, ω), as shown in Eqs 5 and 6:

$$\frac{\partial (\rho k)}{\partial t}+div(\rho k\vec{u})=div(\Gamma \;grad\;k)+S_{k}$$ (5)

$$\frac{\partial (\rho \omega )}{\partial t}+div(\rho \omega \vec{u})=div(\Gamma \;grad\;\omega )+S_{\omega }$$ (6)

where *S*_*k*_ and *S*_*ω*_ are the source terms of k and ω, respectively. In the simulation of the flow of the nozzle to a stationary regiment, the derivatives in time are treated as zero.

**Fig 5:**
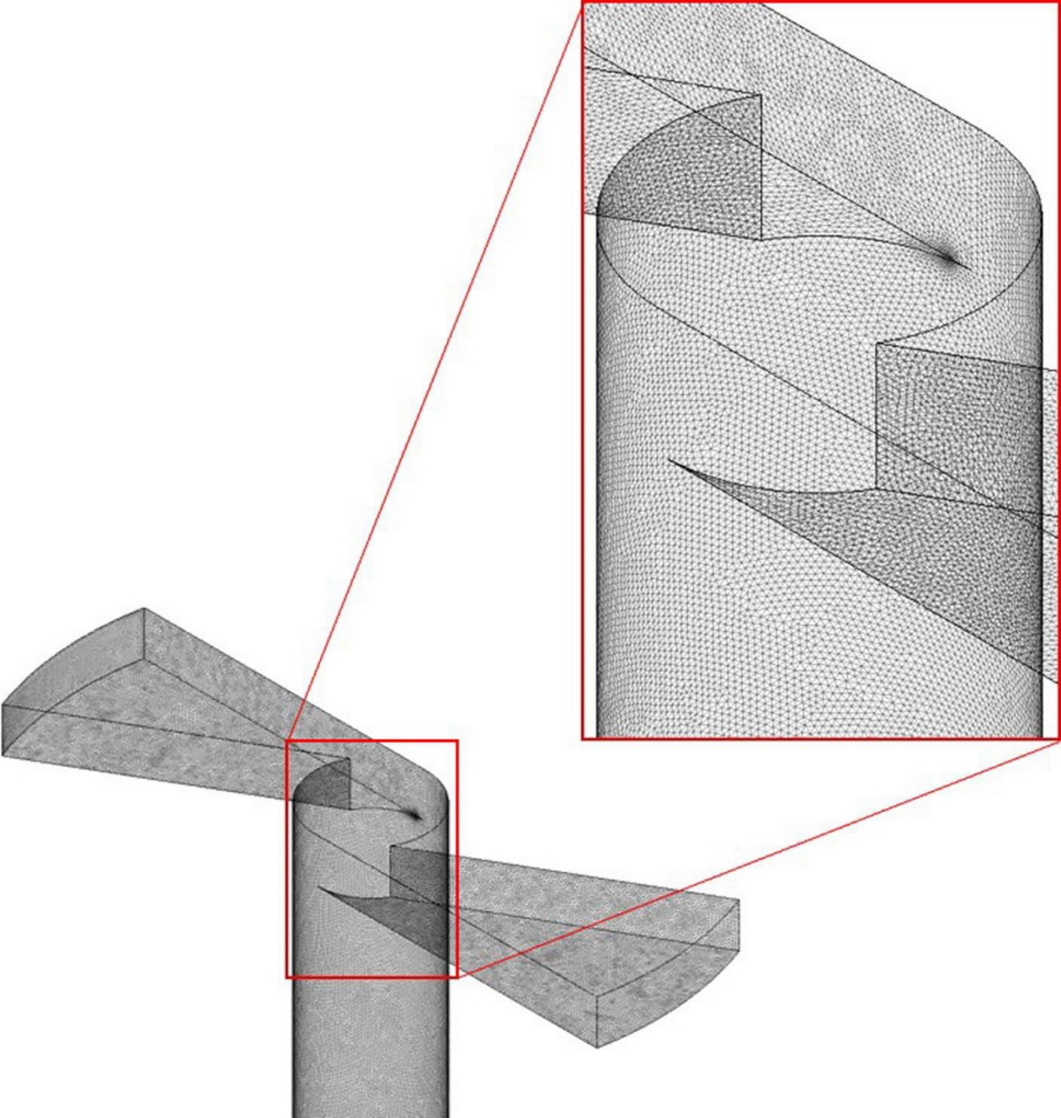
Computational mesh of the nebulizer nozzle.

In order to know if the turbulence model is being used appropriate, it is necessary to evaluate Yplus. The Yplus represents a dimensionless distance from the first node to the wall and, depending on the numerical treatment given to the boundary layer, turbulence models have different ranges suitable for Yplus values. The standard k- ε model, for example, requires an Yplus value on the wall between approximately 30 to 300. According to Salim and Cheah [66] for Yplus values below 30, the k—ε turbulence model is not suitable, being the SST k—ω model more appropriate, as it is a hybrid model between ok—ε and K–ω [67].Once the nozzle computational domain was modeled, it was discretized into control volumes for the application of the discretized governing equations. Fig 5 shows the computational mesh of the simulated geometry with 8,856,031 elements composed of tetrahedrals and pyramids.

For the developed dispersion chamber, it is possible to use different types of products with biocidal properties as well as different concentrations (for example, percentage of dilution in water). For this study, all experiments were performed using sodium hypochlorite at concentrations up to 0.25% as a biocidal agent [68]. Sodium hypochlorite is considered to be one of the most relevant and prevalent disinfectants for disinfecting surfaces against SARS-CoV-2 [69]. For the proposed numerical model, the physical properties were considered as identical to that of water [44]. Thus, they were valid for aqueous solutions of sufficiently low concentrations such that the properties were not significantly impacted. Table 1 shows the boundary conditions (according to the regions named in Fig 4) applied for the simulations with the six nebulizer nozzles in the disinfection chamber. For this study, the flow was considered isothermal and incompressible.

**Table 1:** Contour conditions of the disinfection chamber nebulizer nozzles.

Locations	Boundary Conditions
Inlet	Average flow rates for each nebulizer nozzle
Outlet	Atmosferic Pressure
Wall	No-slip wall

#### Simulation of the disinfection technology (chamber)

**Fig 6:**
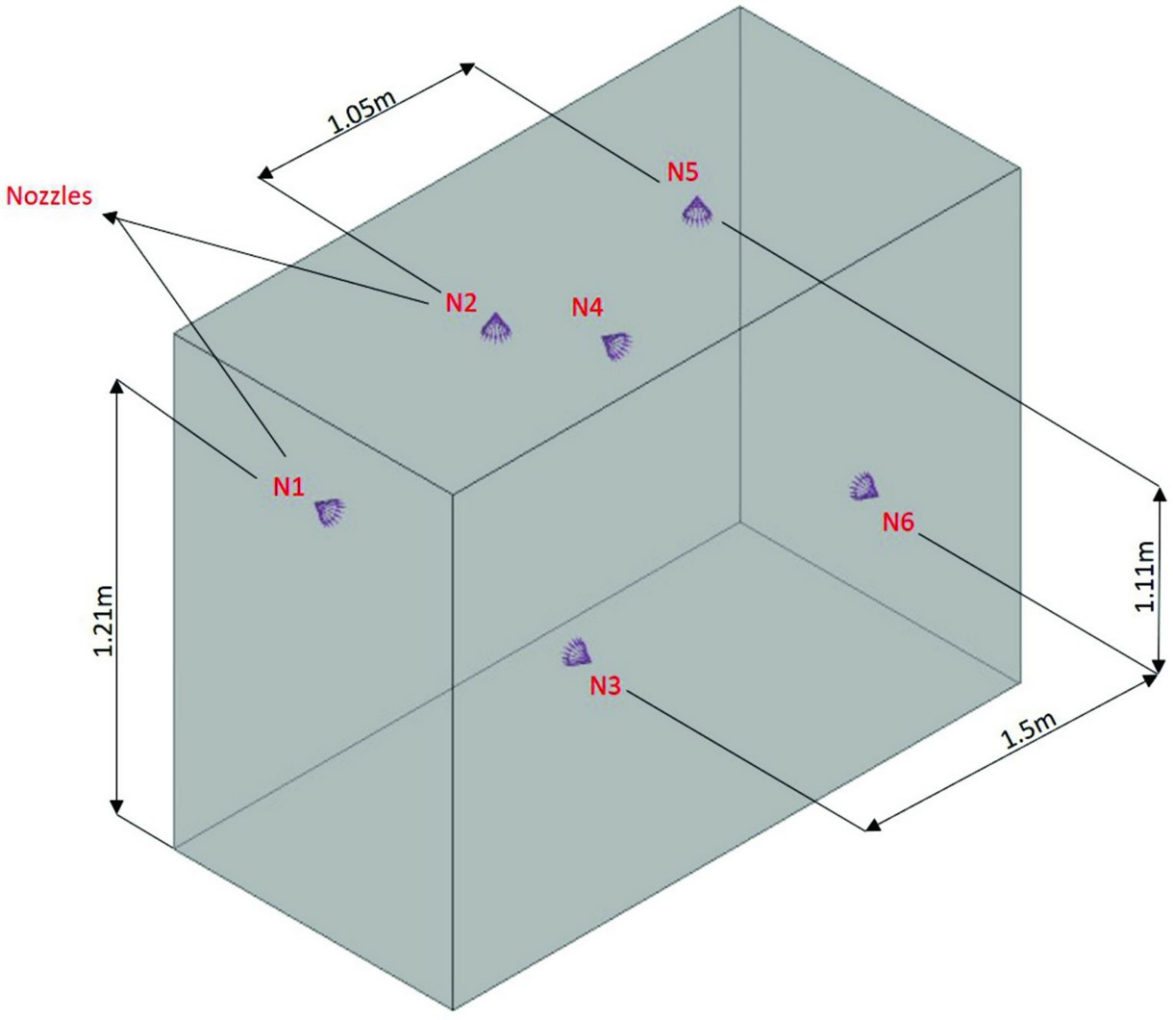
Computational domain of the disinfection chamber.

The disinfection technology (chamber) is represented by a fluid domain that contained the arrangement of the six nebulizer nozzles, as shown in Fig 6. The flow was analyzed through an Eulerian–Lagrangian approach, in which the internal region of the disinfection chamber (continuous medium–ambient air) interacts with the fluid particles (disperse medium) that are generated through the nebulizer nozzles [70, 71].

For the simulation of the disinfection chamber, the continuous medium (air) was treated with an Eulerian approach, using the same mass and moment conservation equations previously presented (Eqs 1–4) but in a transient regime. The biocidal agent solution was treated using a Lagrangian approach as a dispersed fluid (droplets). In this approach, the resulting force on each particle is the sum of the drag (*F*_*D*_) and thrust (*F*_*B*_) forces due to gravity [72]. The particle trajectory can be described according to Eqs 7 and 8:

$$F_{p}=F_{D}+F_{B}$$ (7)

$$m_{p}\frac{\partial v_{p}}{\partial t}=\frac{1}{2}*C_{D}\rho_{f}A_{p}(v_{f}-v_{p})|v_{f}-v_{p}|+\frac{\pi }{6}*d_{p}^{3}(\rho_{p}-\rho_{f})*g$$ (8)

where f indicates the value for the fluid, p indicates the value for the particle, *m* is the mass, *C*_*D*_ is the drag coefficient, *ρ* is the density, *A* is the frontal area, *v* is the speed, *d* is the diameter, and *g* is the gravity.

The drag coefficient is calculated using the Schiller–Naumann model [73], according to Eq 9:

$$C_{D}=max(\frac{24}{Re}*(1+0.15{Re}^{0.687}),0.44)$$ (9)

where *Re* is the Reynolds number.

Three meshes were evaluated for the disinfection chamber with different number of elements (approximately 6.6x10^5^, 1.3x10^6^, and 3.0x10^6^). Each nozzle was considered as a punctual injection of droplets, with mass flow rates defined according to the experimental results. The speeds were set such that they maintained the moment flow resulting from the nozzle simulations. The nozzle dispersion angle value was adopted as determined experimentally. The adopted particle size distribution was described by adjusting the Rosin–Rammler distribution function, which was also evaluated experimentally. All disinfection technology walls were considered “no-slip” with restitution coefficients equal to zero.

### Ethics approval and consent to participate

This study was conducted after its approval by the Ethics and Research Committee of University Center SENAI/CIMATEC (No. 4,132,735), and by following the ethical principles and a written informed consent was obtained.

For this study, only one volunteer was recruited to perform the WSP tests. During the tests, the participant was properly dressed, using all PPE. To avoid contact of the biocidal agent with mucous membranes and skin, as well as to avoid inhalation of the spray formed, the participant used all PPE, including N95 mask, goggles, waterproof coat, gloves, protective shoe and cap.

The participant was followed throughout the study on any type of adverse event. The participant did not manifest or reported any adverse effects related to the use of the disinfection chamber and had no previous allergy to the tested biocidal agent.

The consent form was read and signed by the study volunteer. In addition, participant was also made aware of possible risks, such as the occurrence of an adverse event during or after the use of the chamber, and the benefits, such as reducing the SARS-CoV-2 spread rate.

## Results and discussion

### Spray characterization

Table 2 shows the flow rates for each nozzle and their respective standard deviations. The maximum relative deviation is 3.34%, showing that the individual measured values are slightly different from the average.

**Table 2:** Flow rates of the nebulizer nozzles.

Flow rates Nozzles [L/h]						
Measurement	Nozzle 1	Nozzle 2	Nozzle 3	Nozzle 4	Nozzle 5	Nozzle 6
M1	5.58	5.70	7.50	7.80	5.52	6.72
M2	5.70	5.64	7.50	7.68	5.40	6.84
M3	5.70	6.00	7.50	7.74	5.34	6.90
Average	5.66	5.78	7.50	7.74	5.42	6.82
Standard deviation	0.07	0.19	0.00	0.06	0.09	0.09
	1.22%	3.34%	0.00%	0.78%	1.69%	1.34%

**Fig 7:**
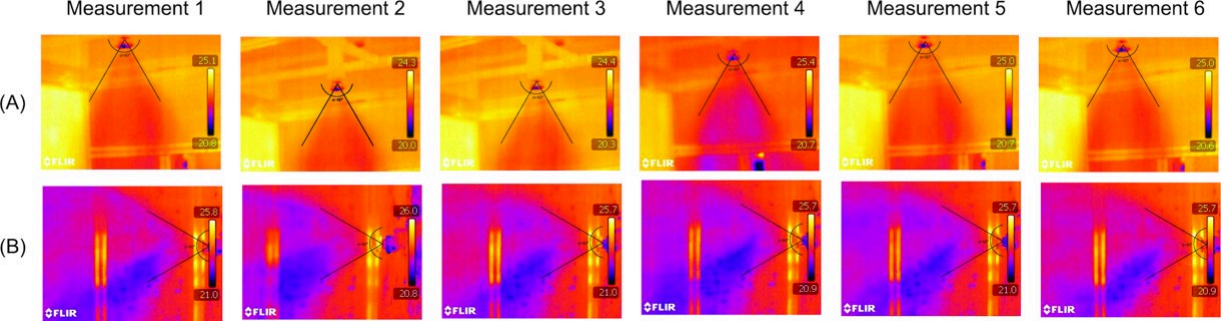
Measurement of the angle formed by the spray generated in the nebulizer nozzles. (A) Vertical position; (B) Horizontal position.

The angle formed by the spray in the two positions is 60°. The captured thermal images (Fig 7) show profiles similar to the characteristic angle.

Bian et al. [74] performed experiments with the orientation angles of the nebulizer nozzle varying from 0° to 90°. The spray cone angles measured through images varied between 60° and 62°, showing that the angle formed by the spray was not affected by the nozzle orientation angle [74].

**Fig 8:**
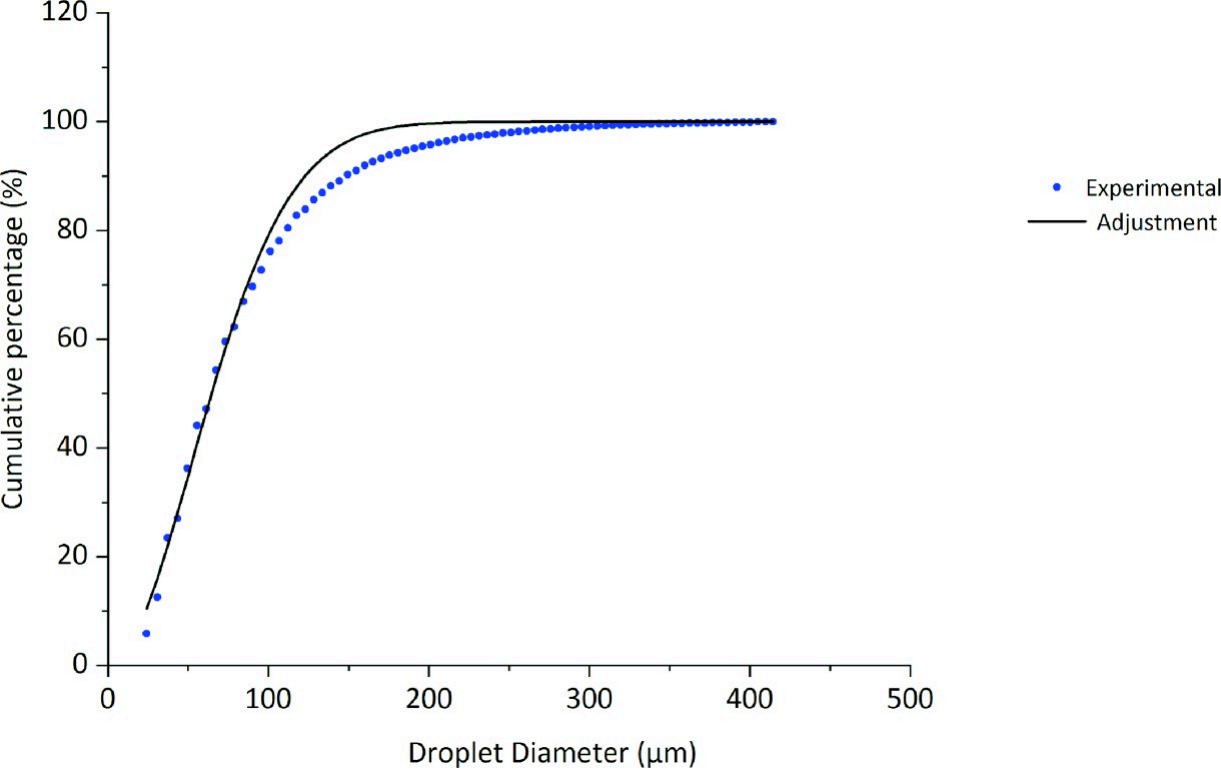
Rosin–Rammler distribution function for the data collected from the WSPs.

Fig 8 shows the cumulative particle size distribution (average of the data collected from the WSPs) and the Rosin–Rammler distribution function curve with *d*_*e*_ = 78.6 *μm* and *γ* = 1.87. The adjusted curve shows a coefficient of determination *R*^2^ = 0.998, characterizing that the adjustment is valid for the representation of the experimental data.

### Wettability analysis

**Fig 9:**
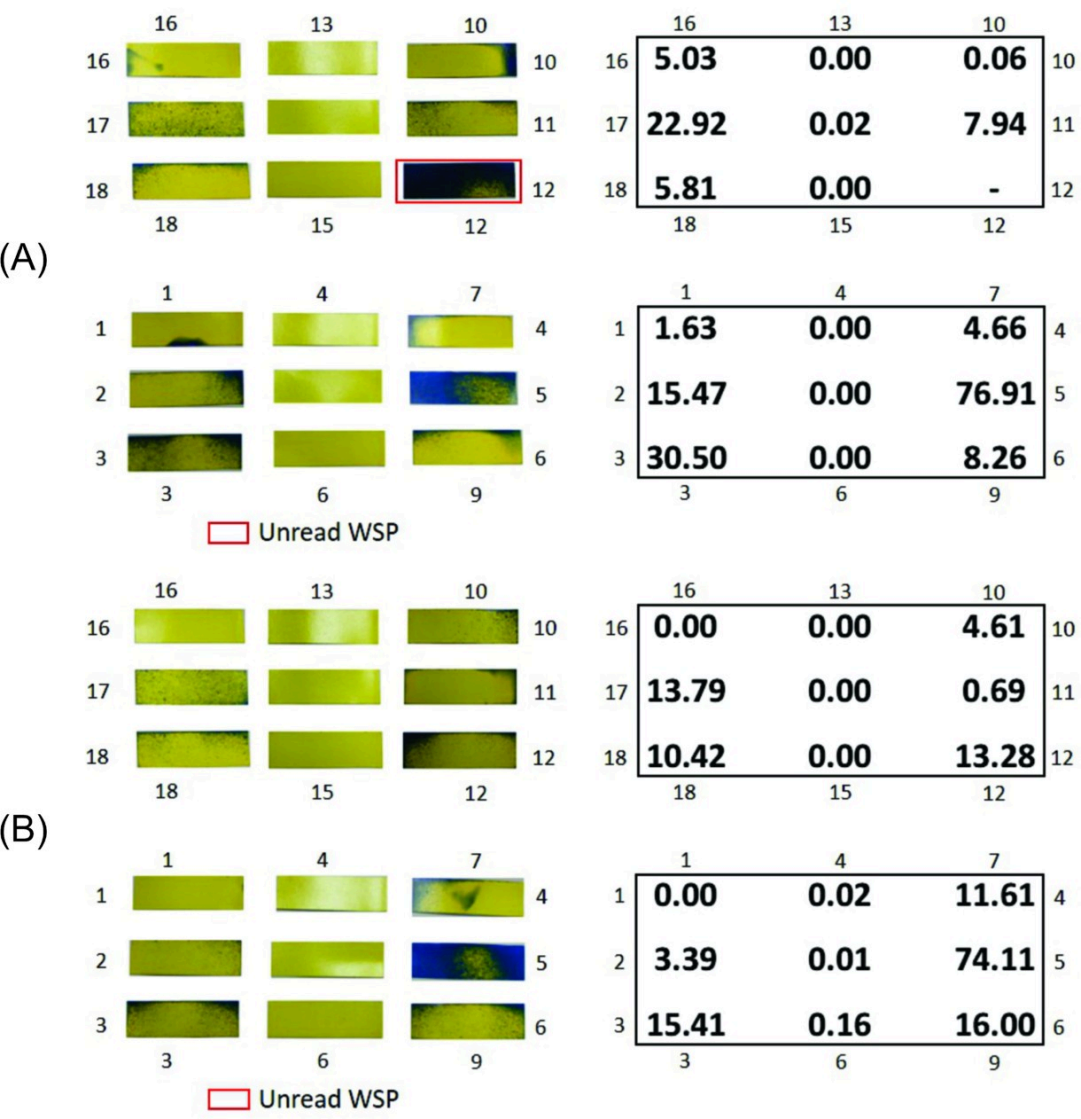
WSPs exposed to the biocidal agent for 10 s and their percentage wet area values. (A) Sample 1; (B) Sample 2.

The samples of the experiment carried out with WSPs applied to the three ranges inside the disinfection chamber and exposed to the biocidal agent for 10 s are shown in Fig 9. The percentages of the wet area for each WSP are also shown.

In the two samples, the areas affected by the solution of the biocidal agent were similar, showing that the method used for evaluation is acceptable. It should be noted that it was not possible to read the WSP with number 12 from sample 1 (Fig 9A). When comparing the WSPs numbered 12 of the two samples, a large difference is visually observed in the area affected by the biocidal agent during exposure. Thus, for this study, the WSP 12 obtained for sample 1 is not considered in the qualitative and quantitative analyses.

In range 2, corresponding to the central region of the disinfection chamber, no significant deposition of particles is observed. Therefore, this can be considered as a limitation of the functionality of the developed equipment and may compromise its effectiveness, regardless of the biocidal agent used (Fig 9).

In a study carried out by Joshi [44], it was observed (side view of the chamber) that even after 12 s of disinfectant dispersion, at regions close to the ends of the structure, the concentration of droplets in the suspension was lower than that in the regions close to the nebulizer nozzles. Thus, it can be guessed that if Joshi [44] experimentally evaluated the dispersion of droplets using the WSP method, the results would be close to those of the samples presented in Fig 9. However, the central region would have a higher concentration of suspended droplets in relation to the ends of the developed chamber.

**Fig 10:**
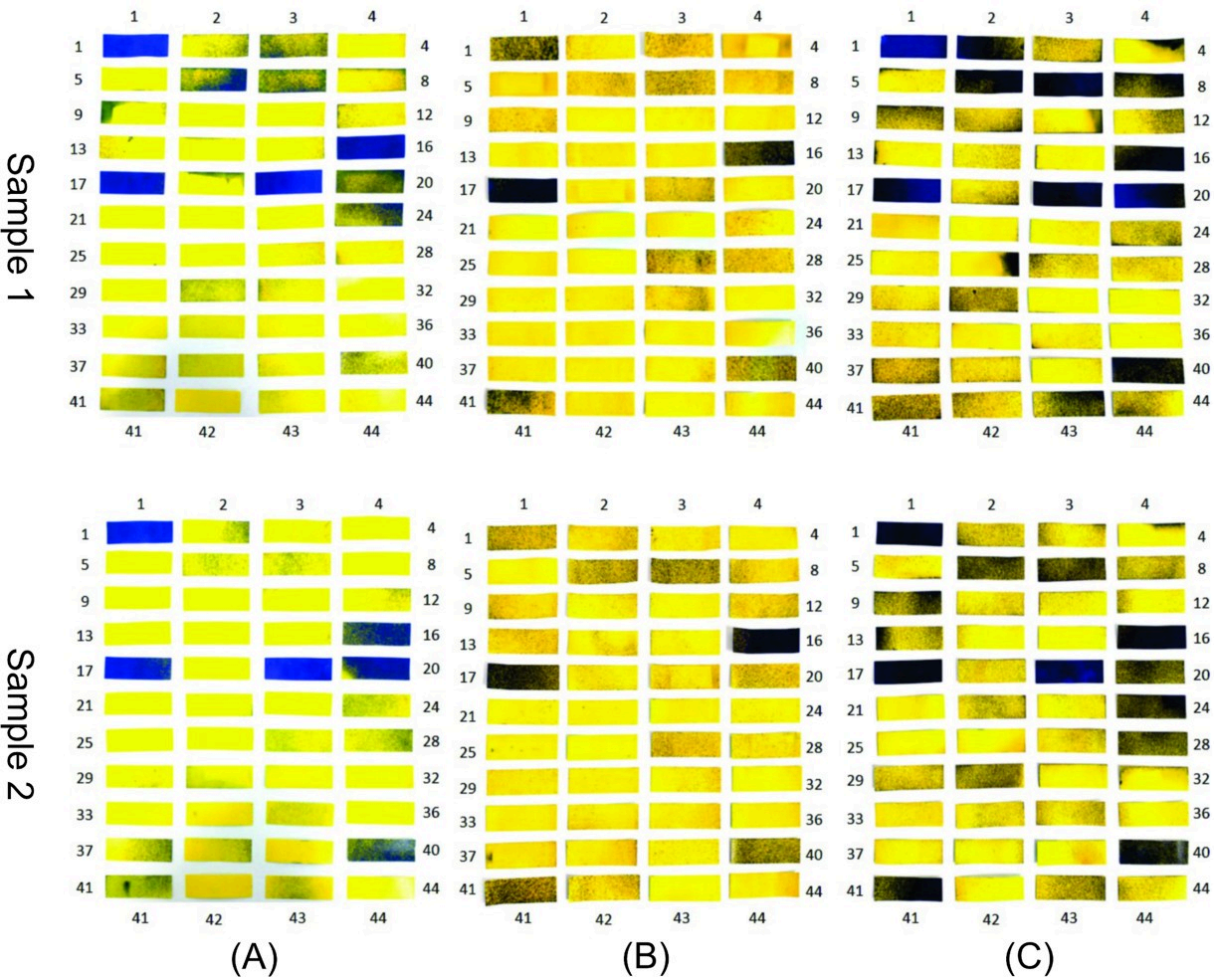
WSPs applied to the body of an individual properly dressed in PPE and exposed to the biocidal agent in the disinfection chamber composed of six nozzles. (A) Exposure for 10 s without turning in the central area of the chamber; (B) exposure for 10 s with 360° rotation in the central area of the chamber; (C) exposure for 30 s with 360° rotation in the central area of the chamber.

### Simulation

**Fig 11:**
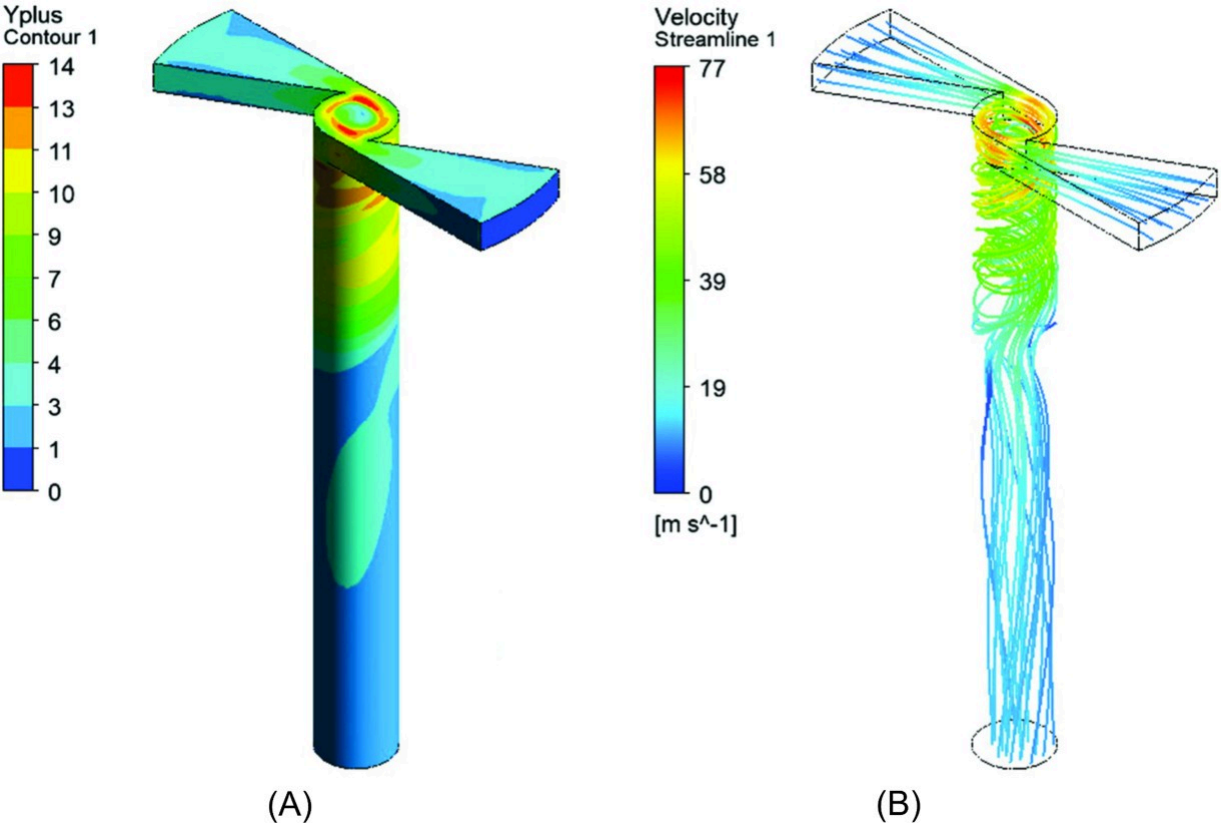
Contours of the nebulizer nozzle. (A) Yplus; (B) Streamline.

Fig 11A illustrates the Yplus contours for the walls of the simulation for the nebulizer nozzle and represents the dimensionless distance to the first computational node. The maximum value of Yplus is 14, thus validating the methodology used for the treatment of the turbulence. This is because the use of the SST model presents good solutions in the treatment close to the wall for a wide range of Yplus [67].

Fig 11B shows the representation of the current lines of flow inside the nebulizer nozzles. It is observed that, owing to the geometric characteristics of the nebulizer nozzle, the highest speeds occur when the flow is redirected to the circular section outlet, causing “swirl” zones.

Table 3 shows the results obtained for the speeds with each average flow of the nebulizer nozzles, which are the boundary conditions for the simulation. The Reynolds number (Re) assumes a minimum value of 4,484 and a maximum value of 6,403, varying according to the average flow of the nozzles.

**Table 3:** Flow rates and velocities of the fluid at the exit of the disinfection chamber nebulizer nozzles.

	Nozzles					
	1	2	3	4	5	6
Guidance	Horizontal	Horizontal	Vertical	Horizontal	Vertical	Horizontal
Mass Flow (g/s)	1.57	1.61	2.08	2.15	1.51	1.89
Velocity (m/s)	35.5	33.9	42.6	49.1	35.8	44.5

**Fig 12:**
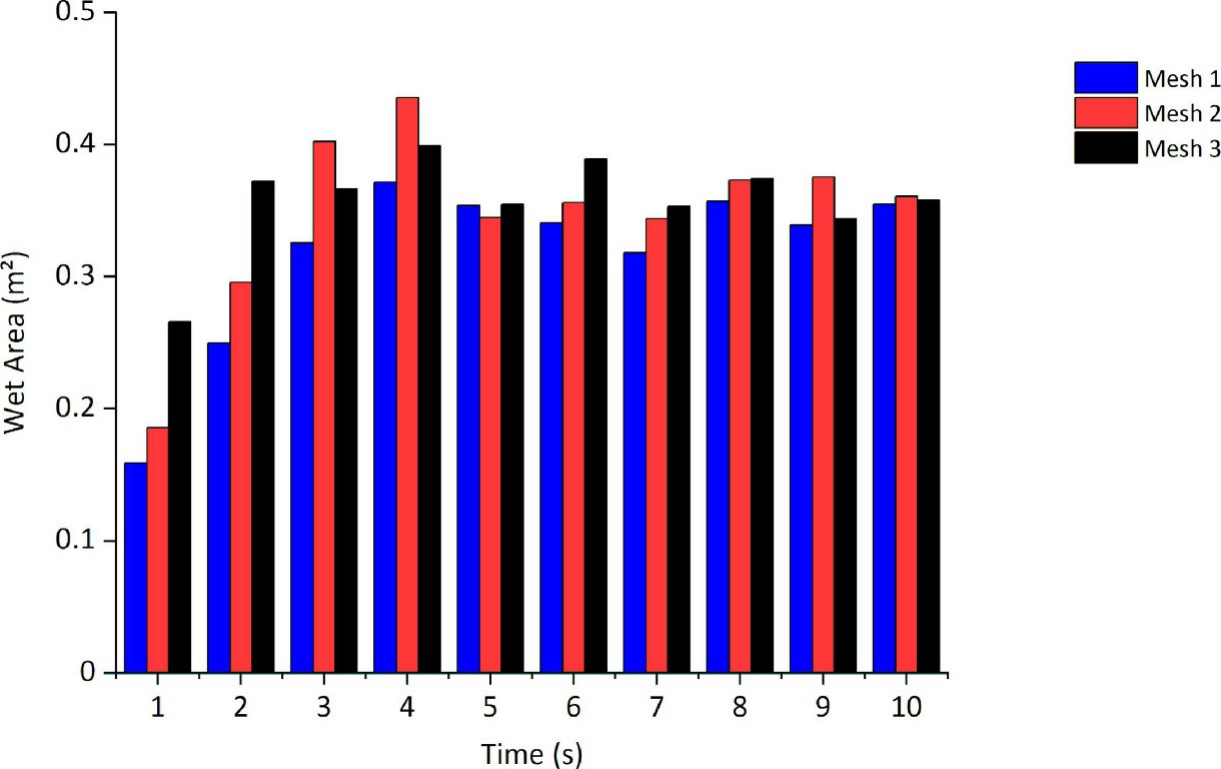
Mesh convergence study.

To analyze the mesh convergence (an important parameter for the stability of the simulation), the wet area results were used in the simulation, along with varying the mesh refinement. Fig 12 shows the results of the wet area in the central plane of the studied device for three different meshes at each instant of time up to a total of 10 s. Meshes 1, 2, and 3 have approximately 6.6x10^5^, 1.3x10^6^, and 3.0x10^6^ elements, respectively. The average relative discrepancy between the results of meshes 2 and 1 is 9.38%, and that between meshes 3 and 2 is 9.36%. Hence, we used mesh 2 to obtain a good relationship between processing time and accuracy.

The injection of particles can occur in two atomization regimes. These include primary atomization, which occurs in the initial conditions for the droplets that leave the nozzle, and secondary atomization, which occurs when the external forces act on the droplets and generate smaller droplets. The secondary rupture of the particles occurs when their Weber number is greater than the critical Weber number [73]:

$${We}_{crit}<We$$

For the evaluation of the maximum Weber number, Eqs 10–12 are used.

$${We}_{max}=\frac{\rho_{ar}*V_{relmax}^{2}*d_{pmax}}{\sigma }$$ (10)

$${We}_{crit}=12*(1+1,077*{Oh}^{1,6})$$ (11)

$$Oh=\frac{\mu_{solução}}{\sqrt{\rho_{solução}*\sigma *d_{p}}}$$ (12)

where *ρ*_*ar*_ is the specific mass, *V*_*relmax*_ is the maximum relative speed, *d*_*pmax*_ is the maximum droplet diameter, *σ* is the surface tension, and Oh is the Ohnesorge number.

In the calculation of the critical Weber number (*We*_*crit*_), only positive values greater than 12 can be achieved. However, in the calculation of the maximum Weber number, consideration of the maximum values for velocity *V*_*rel*_ and droplet diameter *d*_*p*_ results in a value of 7.12, thereby meeting the criterion of no secondary rupture.

**Fig 13:**
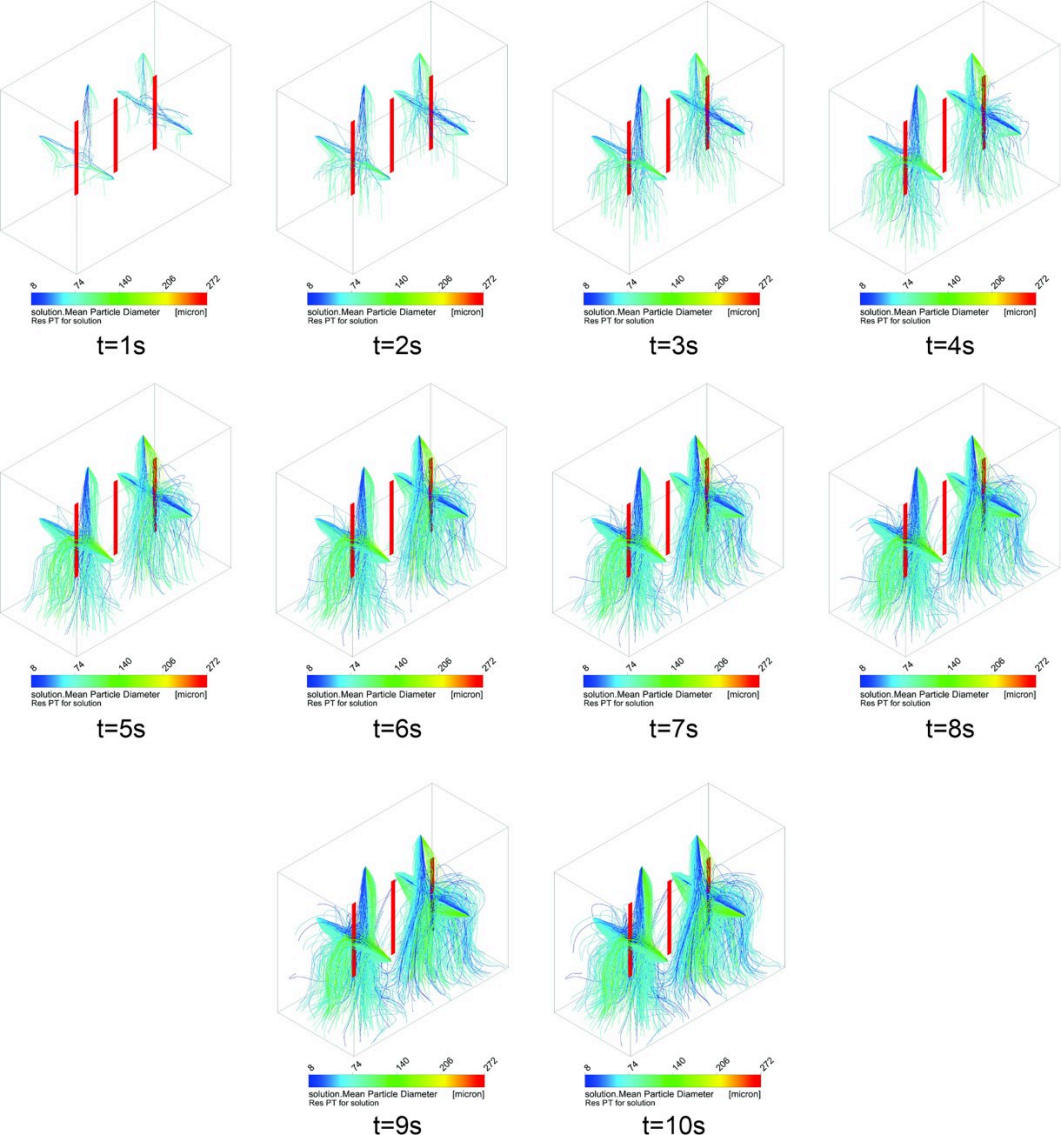
Dispersion of the droplets generated by the six nebulizer nozzles in the disinfection chamber during the exposure time of 10 s.

Fig 13 shows the dispersion of the particles over a time of 10 s for the current configuration of six nozzles of the disinfection chamber. As previously described, the intensity of the flow of droplets is evaluated in the ranges 1, 2, and 3 (Fig 3A), and it is observed that the intensity of the flow increases up to 10 s (Fig 13). It is verified that, in the region of range 2, even within the time of 10 s, there is insignificant concentration of suspended droplets. From the results, a new procedure is proposed for the passage/use of the disinfection chamber, where the need for 360° rotation is modified compared to the central region of the chamber (more specifically in range 2). Thus, based on the simulation results, a new method is proposed with two 360° turns in two different regions of the disinfection chamber (with six projected nozzles). The turning positions should be fixed in the regions with the highest volume of droplets dispersed inside the chamber, i.e., in the regions identified as ranges 1 and 3. It is noted that the droplets that hit the ground at t = 9 s and t = 10 s (Fig 13) stop recirculating within the environment. This occurs because the restitution coefficients are zero, similar to the physical phenomenon in which droplets adhere to solid surfaces [75, 76].

In another study, Joshi [44] presented the results of CFD simulations based on 12-s dispersion times within the developed chamber. However, it was not informed whether the droplets that touched the solid walls of the structure or the floor were discarded. This information is important because, in practice, when a droplet touches a solid surface, it is not dispersed in air (for negligible air velocity on the surface). Without this consideration, the result of the dispersion of droplets in a certain time may be erroneous because some droplets may hit solid surfaces and no longer recirculate in that region.

### Comparative analysis of simulation and experimental tests

**Fig 14:**
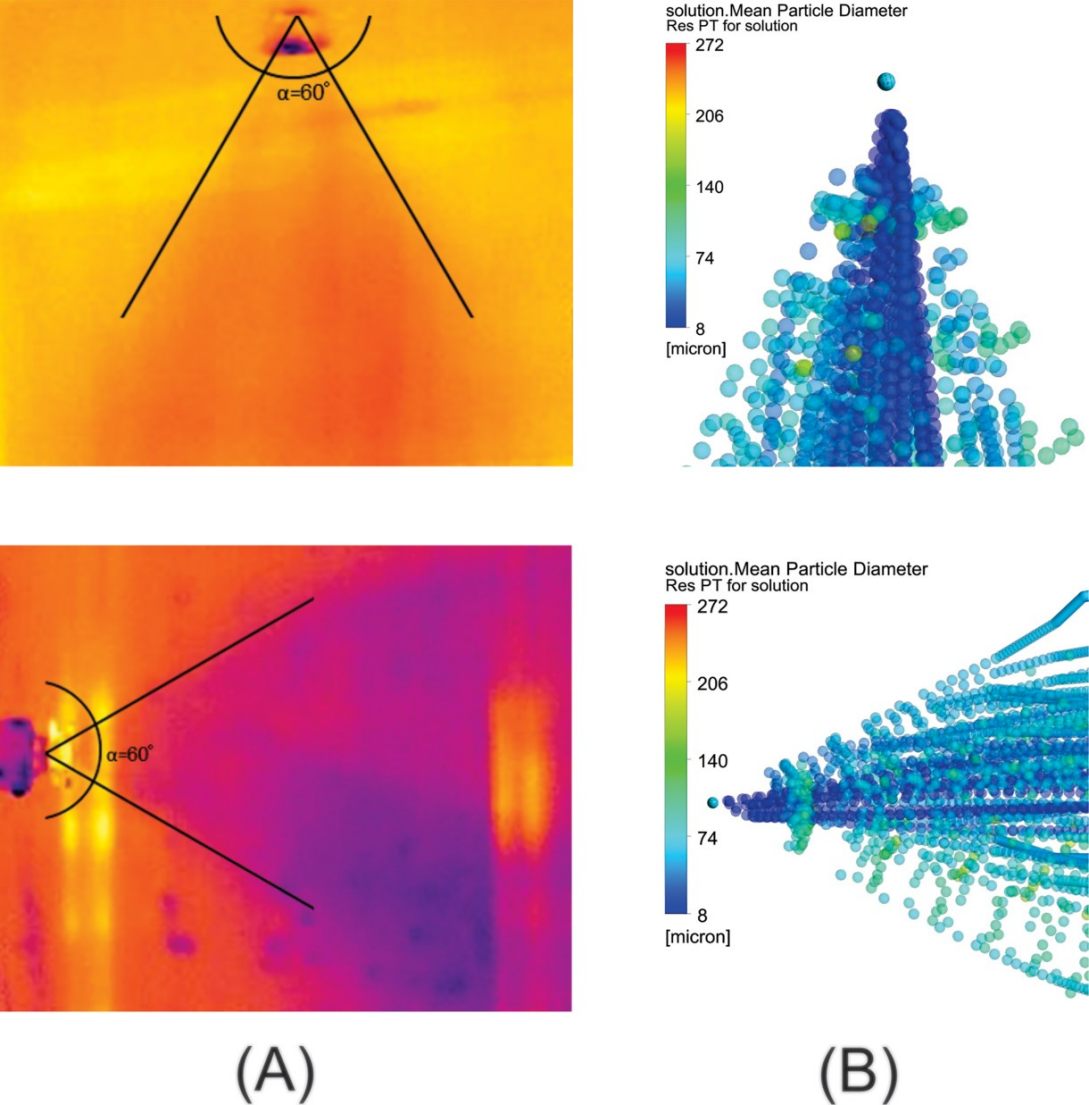
Angle formed by the spray on the nebulizer nozzles in the vertical and horizontal positions. (A) Thermal image; (B) CFD simulation.

**Fig 15:**
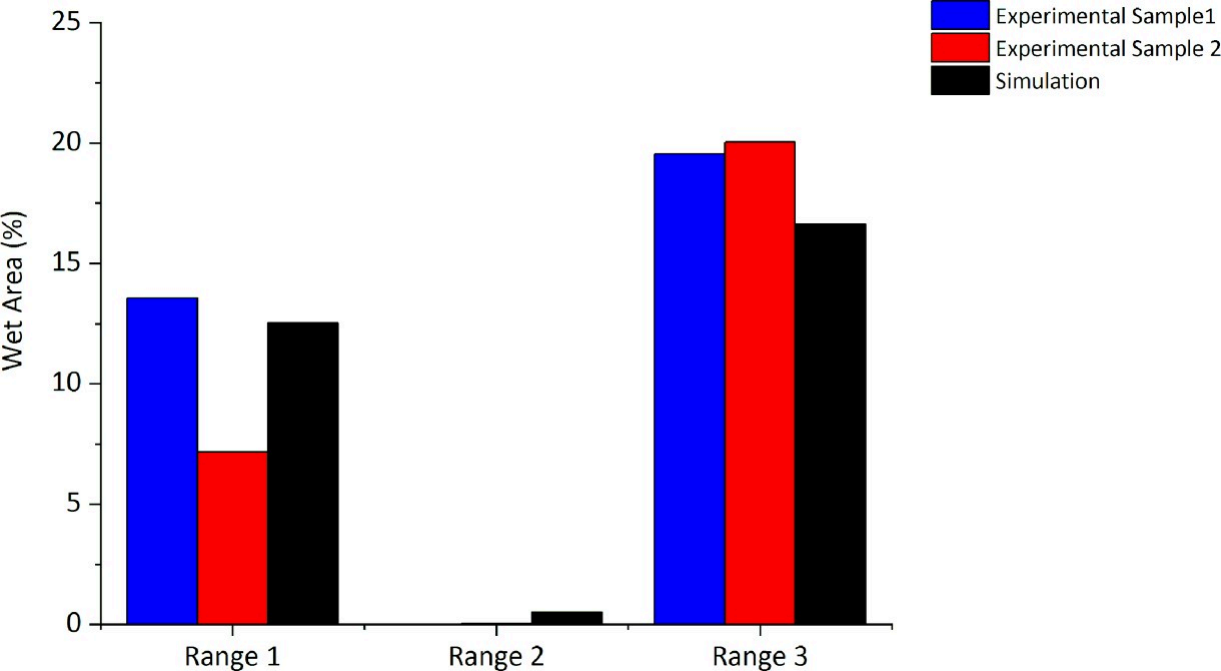
Percentage of wet area in the ranges analyzed inside the disinfection chamber with six nebulizer nozzles (experimental and simulation results).

**Fig 16:**
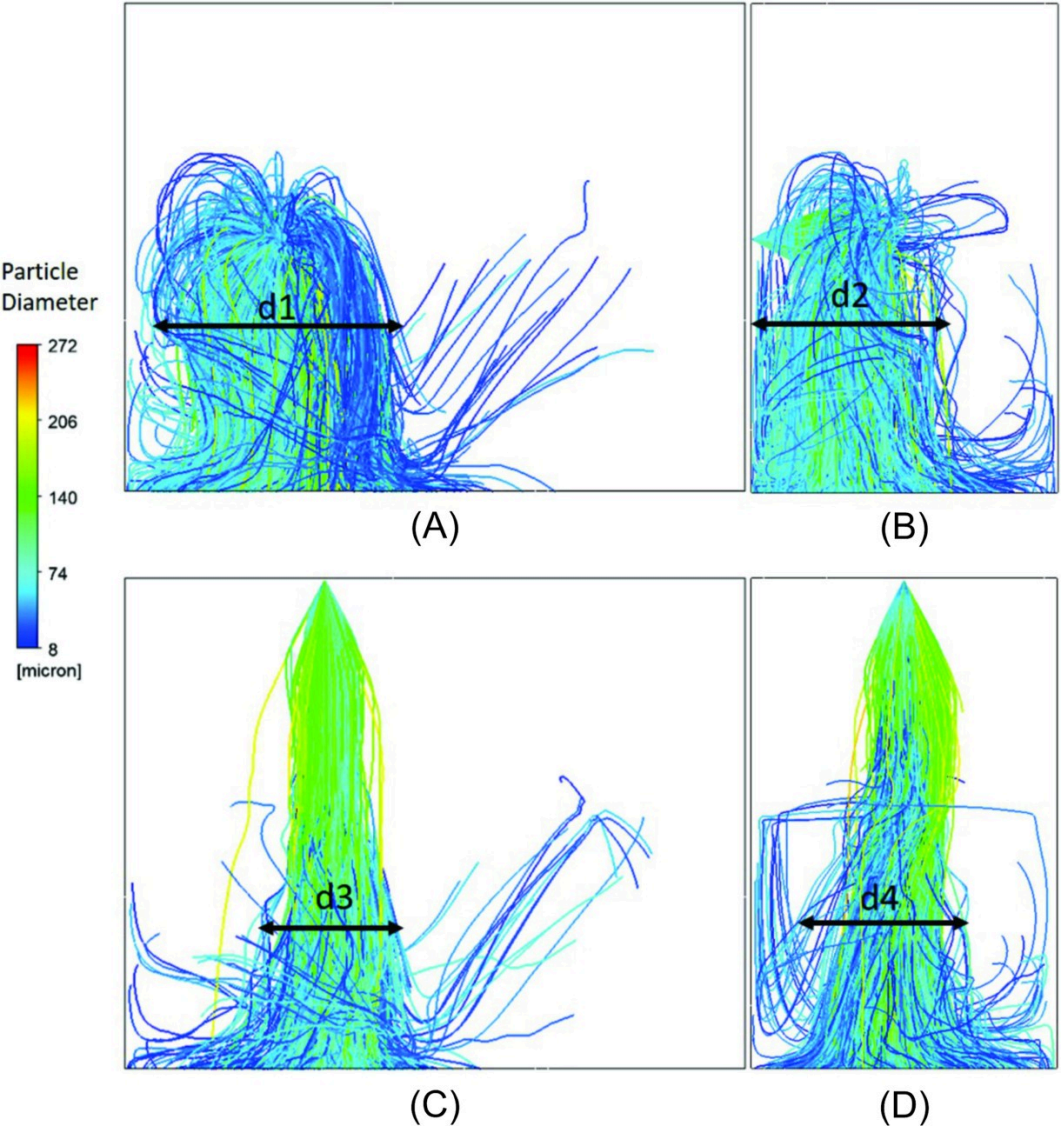
Contours of the nozzles N1 and N2. (A) Front view and (B) Side view of the horizontal nozzle (N1); (C) Front view and (D) Side view of the vertical nozzle (N2).

The angle of the spray formed by the nebulizer nozzle in the simulation shows a behavior close to the angle measured in the experiment, as shown in Fig 14. In Fig 15, the average percentages of the wet area in each of the three ranges are shown, according to the experimental results (Fig 9) and the percentages of the numerical simulation. The results show an agreement between the experiment and simulation. Once again, it is identified that in range 2, there is no dispersion of droplets and that the greatest dispersion occurs in the region of range 3. The experimental results thus confirm that the simulation yields results that identify the need for proposals to achieve improvements in the configuration of the disinfection chamber for better performance, which is the objective of this study. In Fig 16, the dispersion contours of the droplets for nozzles N1 (horizontal) and N2 (vertical) are shown. Distance d1 (Fig 16A) is greater than d3 (Fig 16C) in the front view, and distance d2 (Fig 16B) is greater than d4 (Fig 16D) in the side view of the camera. A greater dispersion of the droplets for the nozzle is observed in the horizontal position compared to the vertical position. Hence, a new configuration of the chamber is proposed in this study. The nozzles must be positioned horizontally to improve the dispersion of droplets in the environment and consequently the effectiveness of the disinfection chamber.

### Proposed new procedure of using the disinfection chamber

**Fig 17:**
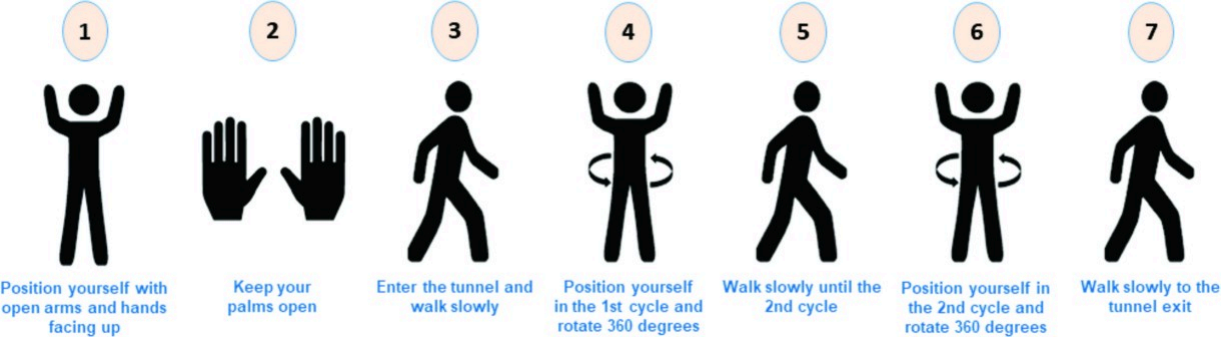
Newly proposed passage procedure for using the disinfection chamber.

From the experimental and simulation results obtained for the dispersion of the biocidal agent solution in range 2 of the previously developed disinfection chamber, a new passage procedure can be proposed, as shown in Fig 17. According to the proposal, the individual performs two complete 360° turns in the regions of ranges 1 and 3, where there is a higher concentration of suspended droplets. Hence, there is greater possibility of the biocidal agent adhering to the surface of the PPE. This can improve the performance of the disinfection chamber, considering that the proposed use is to promote the instant disinfection of PPE before the doffing step.

**Fig 18:**
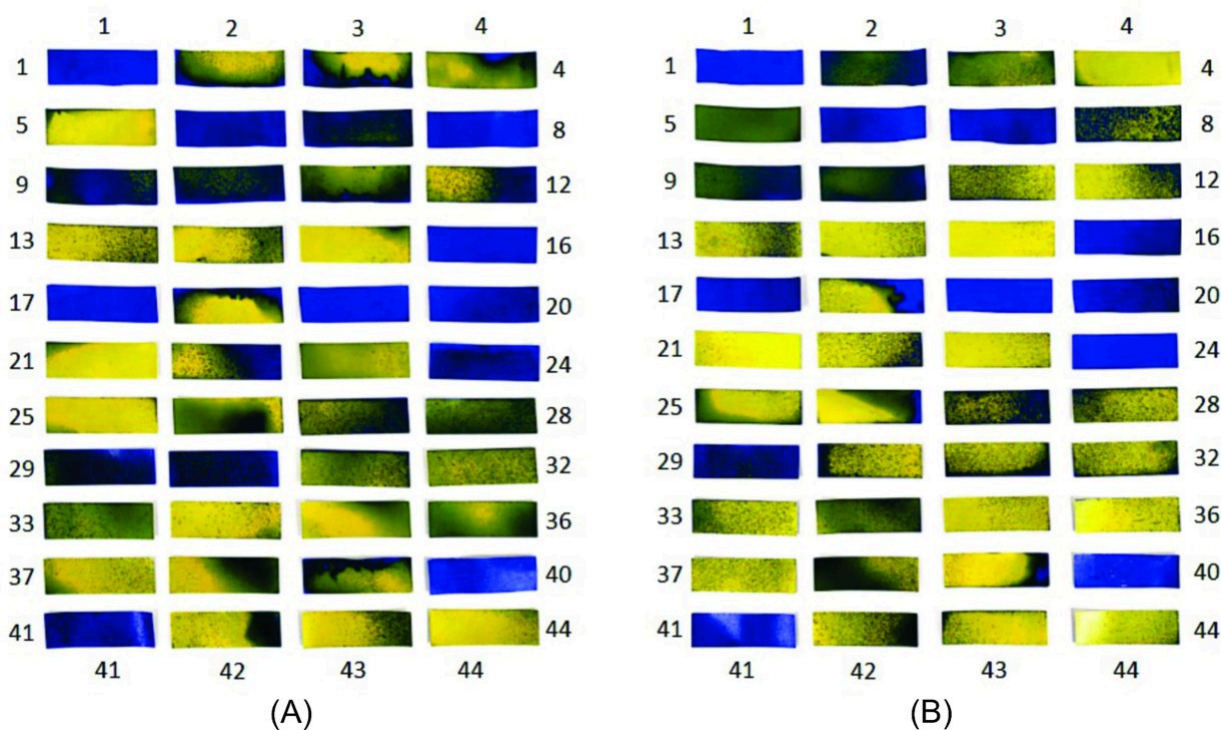
WSPs applied to different regions of the body of an individual properly dressed with PPE and exposed to the biocidal agent during passage through the disinfection chamber with six nozzles in 30 s. (A) Sample 1; (B) Sample 2.

The proposed new procedure was tested experimentally, and improvement was confirmed with an increase in WSP wettability. Fig 18 shows the results obtained for the analysis with the WSPs (two samples from the experiment carried out under the same conditions). From the comparative analysis between the tests performed within the passage procedure with a 360° turn in the central area of the chamber (range 2) (Fig 10C) and with two turns in ranges 1 and 3 of the chamber (Fig 18), the improvement in the use of the disinfection chamber is demonstrated. However, to increase the operational reliability of the system and to ensure that users utilize the technology in the best possible manner, a new configuration for the equipment itself is also proposed with 12 nozzles. It is worth mentioning that the proposed new construction configuration addresses the understanding of the functioning and influence of human factors in human–machine interaction. This is because, in integrated and complex systems, reducing failures requires an understanding of the complexity of human functioning and its cognitive processes [77, 78].

### New proposed configuration for the disinfection chamber

#### Geometry

**Fig 19:**
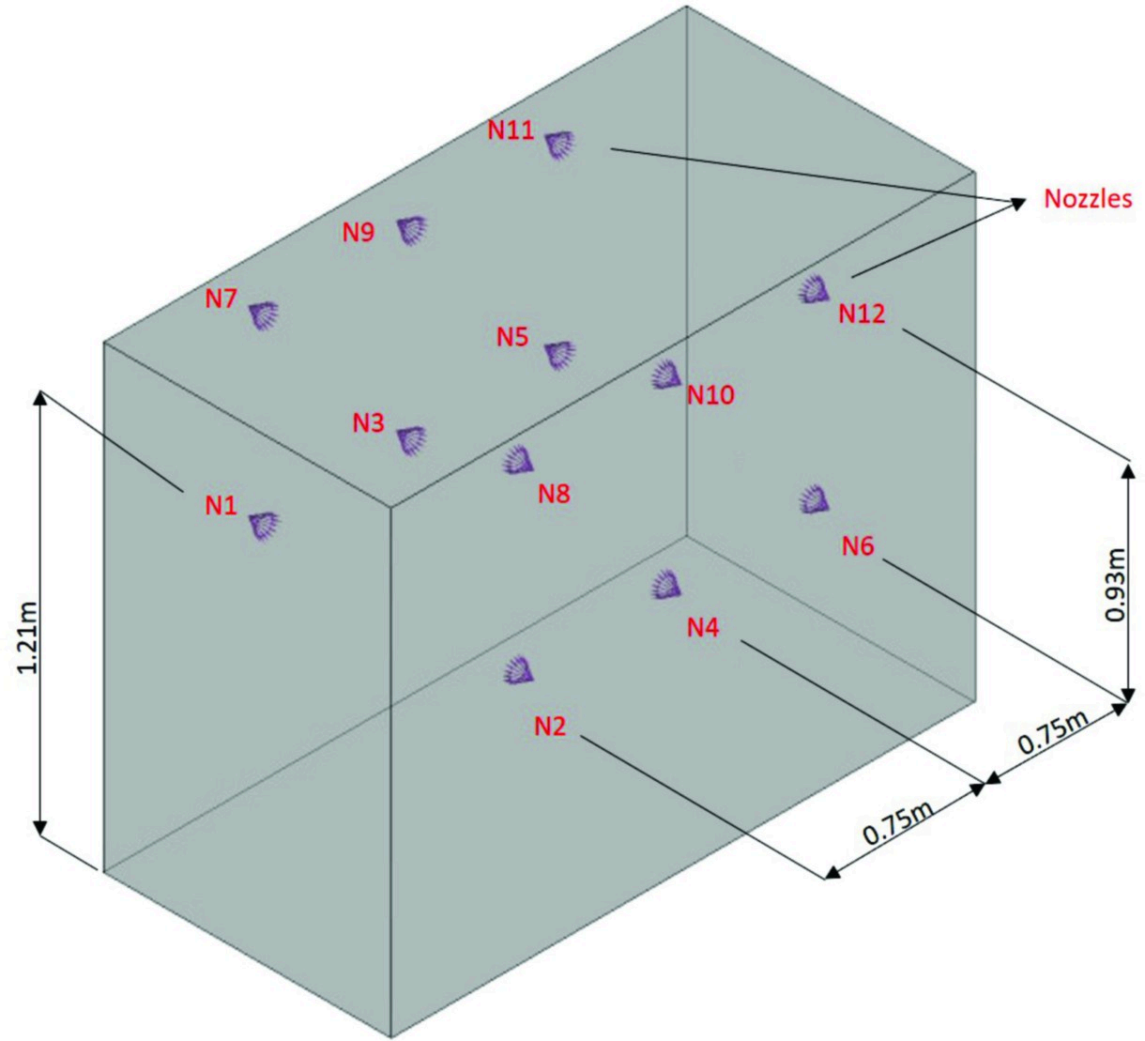
New proposed configuration for the disinfection chamber designed with 12 nebulizer nozzles.

After the simulation analysis, a new configuration is proposed for the chamber, using 12 horizontally mounted nozzles, which allows greater wettability capacity of the exposed area. The new proposed configuration is shown in Fig 19. The wettability allows the uniform and homogeneous action of the biocidal agent in the application of the chamber and its use for instant disinfection of contaminated surfaces. In the proposed configuration, all 12 nozzles are positioned horizontally, with two nozzles at different heights for each side of ranges 1, 2, and 3 (Fig 19). The proposed configuration also aims to minimize possible interference related to human factors. Thus, even if the user passes through the interior of the chamber without making a complete 360° turn in the center of the chamber, the arrangement can ensure that droplets of the biocidal agent solution are deposited on the surface of interest.

#### Simulation

**Fig 20:**
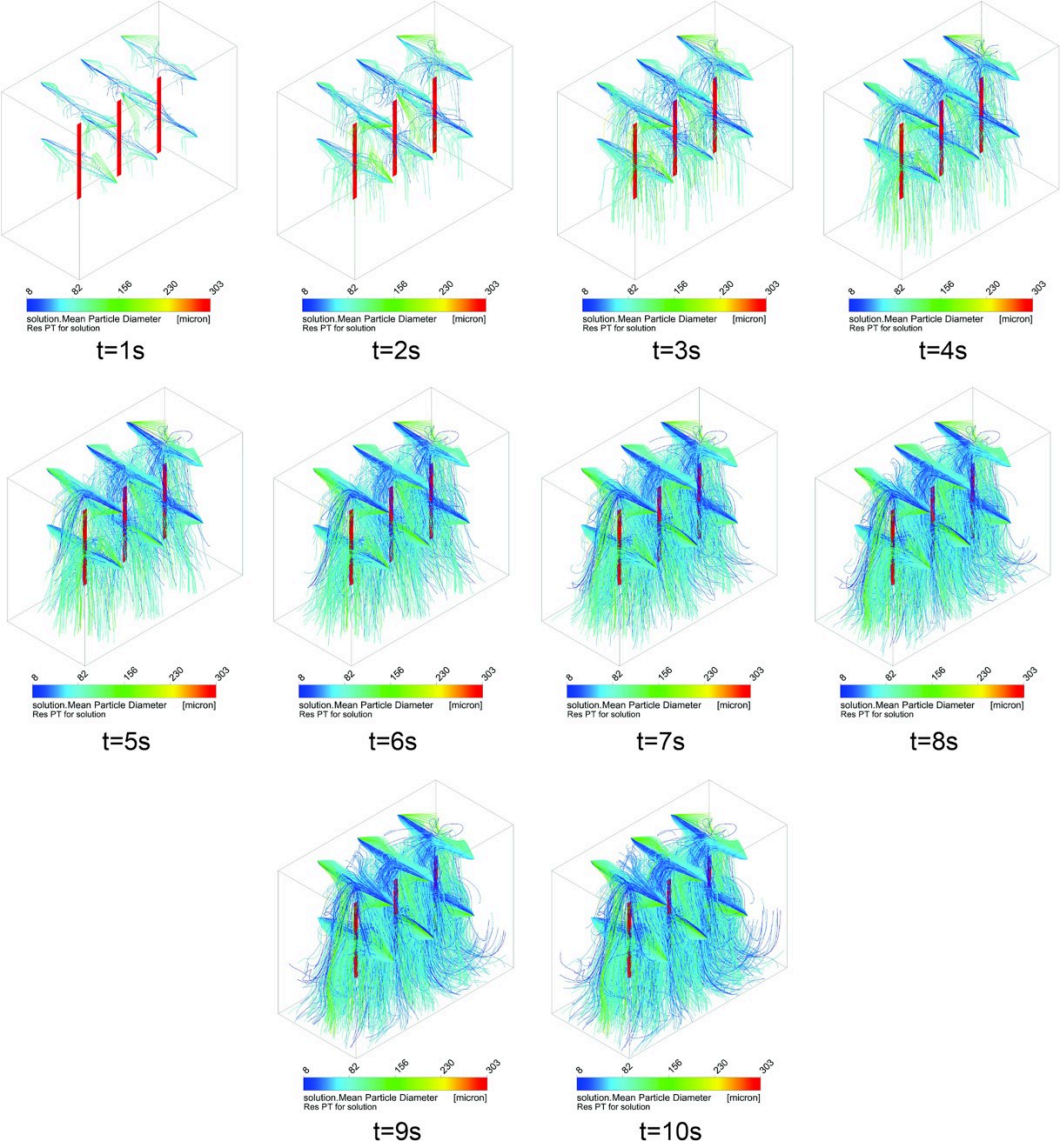
Dispersion of the droplets generated by the 12 nebulizer nozzles according to the new configuration of the disinfection chamber.

Fig 20 shows the dispersion of the solution over time (10 s) for the new configuration proposed for the disinfection chamber, i.e., with 12 nebulizer nozzles. Thus, the intensity of the flow of droplets is evaluated in the ranges 1, 2, and 3 for 10 s, and an increased flow intensity is observed for this new configuration, as compared to the previous configuration of six nozzles. Significantly improved droplet deposition behavior is observed in the region of range 2 (Figs 13 and 20). It is noteworthy that in this configuration, the region of range 2 behaves similar to the ranges 1 and 3, and the suspended droplets are capable of reaching the surfaces of all areas evaluated in this study.

**Fig 21:**
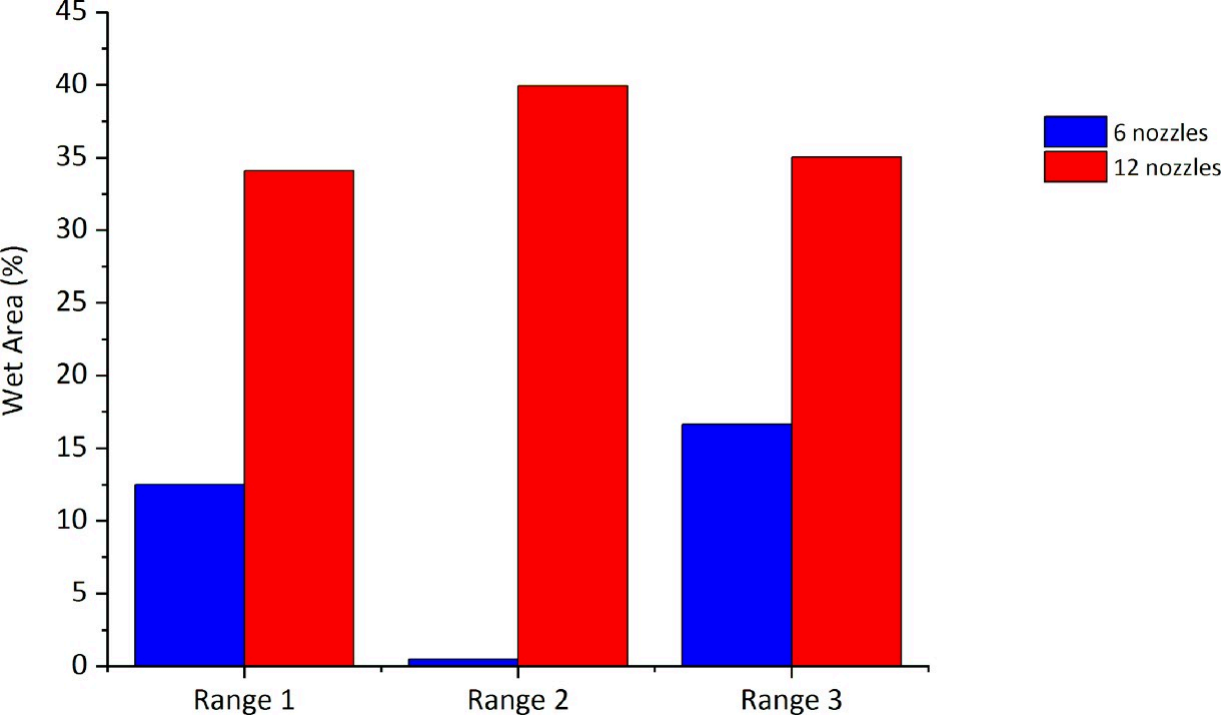
Percentage of wet area in the configurations analyzed for the disinfection chamber with 6 and 12 nebulizer nozzles.

Fig 21 presents the results for the percentage of wet area in the ranges 1, 2, and 3 obtained from the simulations of chamber configurations with six and twelve nebulizer nozzles. The comparative analysis in relation to the percentage of the wet area shows that the proposed new configuration significantly increases the suspended droplet configuration in all the studied ranges. It is important to highlight that, with the new configuration, range 2 represents the region with the highest concentration of suspended droplets (39.94%) (represented by the central area of the disinfection chamber), thus demonstrating the potential for application of the proposed new configuration.

#### Experimental tests

**Fig 22:**
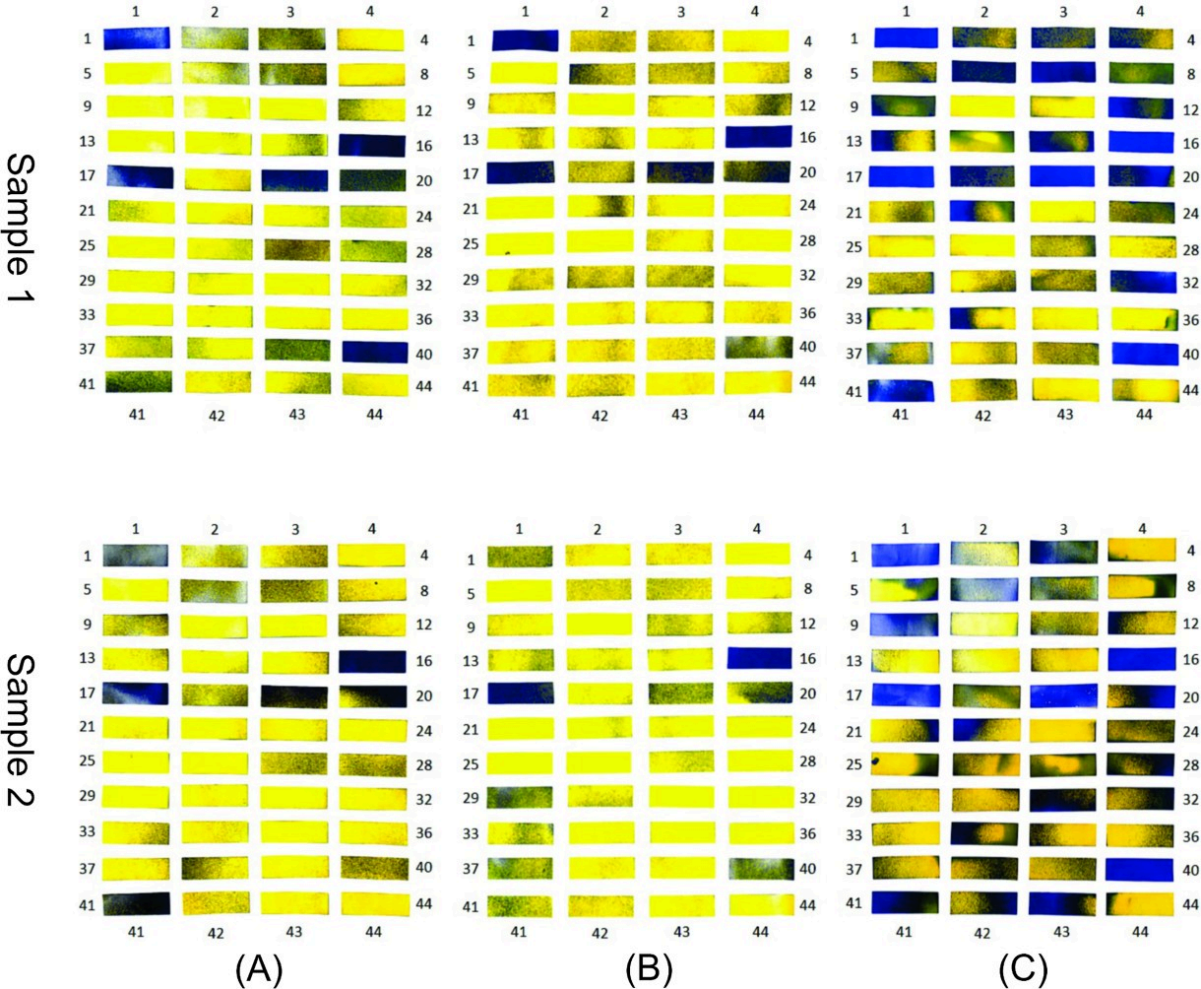
WSPs applied to the body and exposed to the biocidal agent (12 nozzles). (A) Exposed for 10 s without turning; (B) Exposed for 10s with turning in the center; (C) Exposed for 30 s with turning in the center.

To confirm the simulation results with the new configuration, experiments were also carried out to analyze the wettability using WSPs, and the results are shown in Fig 22. From this analysis, a similar wettability pattern is observed. This pattern is justified by WSPs 1, 16, 17, 19, 20, and 40, which wet more intensively in all samples, even with different exposure times and passage procedures. It is also observed that the WSPs exposed to the biocidal agent for 30 s (Fig 22C) have the highest droplet deposition. Another important detail is that, for the same time of 10 s, the WSPs in Fig 22A and 22B show similar wettability.

#### Comparison of wettability between the chambers with six and 12 nozzles

From the comparative analysis between the chambers with six nozzles (Fig 13) and twelve nozzles (Fig 20), the configuration with greater number of nozzles shows better performance with respect to the dispersion of droplets, covering all the equipment. In this way, and as previously mentioned, even if users perform turns in different regions between ranges 1 and 3, greater wettability is achieved when compared to the procedure of turning in the center of the equipment in the configuration with six nozzles. In Fig 21, it is evident that there is a significant increase in the concentration of suspended droplets in the regions between the ranges 1 and 3. In addition, comparing the WSPs of the experimental tests shown in Figs 10 and 22, it is observed that there is an increase in the wet area (blue color) in all experiments. This increase is more significant for the exposure time of 30 s (Figs 10C and 22C). Another important detail is that even though the passage procedure was performed differently (Fig 22A and 22B), the WSPs show similar behavior. This is in contrast to the WSPs obtained for the chamber with six nozzles (Fig 10A and 10B). Thus, with the new configuration, the human factor can be significantly minimized.

This study demonstrated the effectiveness of aspersion the disinfection chamber. Thus, these technical parameters and data found can help subsidize an increase in scale for the equipment tested, in future development studies, since the technical characteristics of construction and application of the equipment were amply explained in this research. This may allow a greater expansion of the application of this equipment through its distribution to other sites, covering a larger number of hospitals, for example, or other areas where the principle of the technology can be applied for the decontamination of potential surfaces.

## Conclusion

The experimental tests were carried out twice and showed similar results, thus demonstrating that the applied methodology is adequate for the proposed study. It is important to say that the evaluation performed was for the passage of one person at a time inside the chamber. However, the possibility of more than one person at a time may be considered in future studies, applying the same evaluation of the new configuration performed in this study to affirm the non-interference in the results by passing through the chamber in a shared manner.

The mesh of the geometry used in the numerical simulations shows good convergence, and its parameters are within the reported range in literature. Comparing the experimental and simulation results of the current configuration, it is concluded that the use of the CFD tool is sufficient to understand the flow behavior within the chamber. A good agreement between the numerical and experimental results was observed. This model can be used to propose new configurations for the disinfection technology.

The mathematical model was validated, and simulations of the proposed new configuration were performed. The simulation results confirm that the proposed configuration increases the wettability of the human body. In this new configuration, it is possible to considerably increase the suspended droplets in ranges 1, 2, and 3. The droplet concentration increases from 12.53% to 34.11% in range 1, from 0.51% to 39.94% in range 2, and from 16.65% to 35.05% in range 3.

The experimental results of the new configuration prove that there is an increase in wettability at all exposure times, and it is more significant for an exposure time of 30 s. Even with different passage procedures, there are no significant differences in the results, thus demonstrating the effectiveness of the new configuration in minimizing the impact of human factors on the disinfection technology.

The present study is limited to the geometry conditions of the disinfection chamber and the operational parameters studied (either through simulation or experimentally). Conclusions about other conditions need to be evaluated since the flow profiles may undergo significant changes.
